# Pasteurized *Akkermansia muciniphila* as a candidate multi-effector pharmacological system in the gut-liver axis: evidence, unresolved causality, and translational priorities

**DOI:** 10.3389/fphar.2026.1956062

**Published:** 2026-09-23

**Authors:** Yifan Liu, Zihao Xu, Dong-Seon Kim, Hai Xu, Huikun Wu

**Affiliations:** 1 Hubei University of Chinese Medicine, Wuhan, China; 2 Hubei Province International Science and Technology Cooperation Base for Innovative Research and Development of Food and Drug Resources and Targeted Delivery, Wuhan, China; 3 Korea Institute of Oriental Medicine, Daejeon, Republic of Korea; 4 Huangjiahu Hospital of Hubei University of Chinese Medicine, Wuhan, China; 5 Hubei Shizhen Laboratory, Wuhan, China; 6 Institute of Liver Diseases, Hubei Key Laboratory of Theoretical and Applied Research of Liver and Kidney in Traditional Chinese Medicine, Hubei Provincial Hospital of Traditional Chinese Medicine, Wuhan, China; 7 Hubei Provincial Clinical Research Center of Traditional Chinese Medicine for Liver Diseases, Wuhan, China; 8 Affiliated Hospital of Hubei University of Chinese Medicine, Wuhan, China

**Keywords:** Akkermansia muciniphila, Amuc_1100, gut-liver axis, MASLD, pasteurized, postbiotic

## Abstract

Pasteurized *Akkermansia muciniphila* is a clinically tractable postbiotic candidate, but its activity cannot be assigned to a single principle. Heat-stable Amuc_1100 is demonstrably retained in pasteurized cells, whereas the GLP-1-inducing protein P9, extracellular vesicles (AmEVs), membrane lipids, and diffusible metabolites are supported mainly by studies of live bacteria or isolated preparations, whose retention and activity in pasteurized products remain unestablished; together they create overlapping links between the intestinal niche and hepatic physiology. Liver-directed studies now extend this framework beyond indirect metabolic inference. In mice, pasteurized bacteria and Amuc_1100 improve steatosis and alcohol-associated liver injury, while AmEV-containing preparations attenuate experimental fibrosis. Acetate, propionate, and L-norleucine associated with *Akkermansia muciniphila* also engage defined hepatic metabolic or antioxidant pathways. Yet the contribution of each effector, its exposure-response relationship, and its strain dependence remain unresolved. Human studies still evaluate whole-cell preparations and report heterogeneous metabolic and weight-related outcomes rather than liver histology or validated MASLD efficacy endpoints, and they lack validated effector-level pharmacodynamic assays. Here, we integrate the evidence, define the principal causal and translational gaps, and identify experiments that could close them. Priorities include orthogonal effector deconvolution, measurement of effector exposure in stored clinical samples, and specifications that link strain and processing to potency. Purified effectors need not replace pasteurized cells. Instead, *A. muciniphila* should be developed as a measurable multi-effector system, allowing product format to be selected according to the indication and the evidence.

## 1 Introduction

Metabolic dysfunction-associated steatotic liver disease (MASLD) affects about one-third of adults and is sustained by interacting metabolic, inflammatory, and microbial processes (Younossi et al., 2016; Younossi et al., 2025). The gut-liver axis links intestinal barrier integrity, microbial products, and hepatic immune-metabolic responses, making microbial therapeutics attractive but pharmacologically hard to define (Tilg et al., 2022; Hsu and Schnabl, 2023). *Akkermansia muciniphila* is prominent here because live, pasteurized, and component-based preparations each produce metabolic or liver-relevant effects, and pasteurized whole cells have already entered human studies (Liu et al., 2026; Depommier et al., 2019; Wu et al., 2020a).

The 2021 International Scientific Association for Probiotics and Prebiotics consensus defines a postbiotic as a preparation of inanimate microorganisms and/or their components that confers a health benefit (Salminen et al., 2021). Here, pasteurized whole-cell *A. muciniphila* refers to the intact heat-inactivated preparation, whereas effector-defined preparations refer to isolated or enriched proteins, extracellular vesicles, lipids, or metabolites. This distinction matters: pasteurization can preserve a distributed set of bioactivities while improving product stability, but the resulting preparation is not pharmacologically equivalent to any one purified molecule.

This narrative Mini Review used a targeted search of PubMed, Web of Science, Embase, and ClinicalTrials.gov, last updated 23 August 2026. Records were identified using combinations of the terms ‘*Akkermansia muciniphila*’, ‘pasteurized’, ‘postbiotic’, ‘Amuc_1100′, ‘P9′, ‘extracellular vesicle’, ‘metabolite’, ‘MASLD’, ‘NAFLD’, ‘MASH’, ‘liver fibrosis’, and ‘gut-liver axis’, together with ‘clinical trial’ for interventional studies. Searches were restricted to English-language publications, with no lower date limit. Records were eligible if they reported *A. muciniphila* preparations (live, pasteurized, or component-defined) or *A. muciniphila*-associated effectors (proteins, extracellular vesicles, lipids, or metabolites) in metabolic or liver-relevant models or in human studies. Titles and abstracts were screened independently by two authors (Yifan Liu and Zihao Xu), and full texts were assessed against these criteria. Table 1 is intentionally selective: it includes studies directly informing the pharmacological translation of pasteurized whole-cell or effector-defined preparations, and omits studies reporting only live-bacteria ecology without an effector- or pasteurization-relevant link. Because this was not a systematic review, statements that no studies were identified are bounded to this search; registered-trial information reflects ClinicalTrials.gov as accessed on 26 August 2026.

**TABLE 1:** Representative evidence linking pasteurized *A. muciniphila* and its effectors to metabolic and liver-relevant outcomes. The table is intentionally selective and emphasizes evidence that informs pharmacological translation. Abbreviations: 16S rRNA, 16S ribosomal RNA; AmEV, *A. muciniphila*-derived extracellular vesicle; CCl_4_, carbon tetrachloride; CFU, colony-forming unit; FIB-4, fibrosis-4 index; FMT, fecal microbiota transplantation; GLP-1, glucagon-like peptide-1; HFD, high-fat diet; HFHFD, high-fat high-fructose diet; HSC, hepatic stellate cell; ICAM-2, intercellular adhesion molecule-2; ITT, intention-to-treat; MASLD, metabolic dysfunction-associated steatotic liver disease; MASH, metabolic dysfunction-associated steatohepatitis; MRE, magnetic resonance elastography; NAFLD, non-alcoholic fatty liver disease; RCT, randomized controlled trial. ClinicalTrials.gov records were accessed on 26 August 2026.

Evidence stage	Study/preparation	Design/model and population	Primary endpoint and result	Sponsorship/COI	Key limitation/translational value
Mechanistic preclinical	Plovier et al. (2017) Pasteurized MucT; Amuc_1100	Diet-induced obese mice; receptor assays	Pasteurization enhanced selected metabolic effects; purified Amuc_1100 partly reproduced whole-cell activity.	—	Foundational sufficiency evidence; not a liver-specific deconvolution study.
Mechanistic preclinical	Yoon et al. (2021) Purified P9	Enteroendocrine cells; HFD-fed mice	P9 bound ICAM-2, induced GLP-1, and improved systemic metabolic phenotypes.	—	No direct histological MASH or fibrosis test; endogenous P9 exposure in pasteurized products unknown.
Liver preclinical	Keshavarz Azizi Raftar et al. (2021) Live, pasteurized, and AmEV preparations	LX-2 cells; HFD/CCl_4_ mouse model	Improved permeability, inflammation, histopathology, and fibrosis-related markers; suppressed HSC activation-associated responses.	—	Preparations were not quantitatively matched by active-effector exposure.
Liver preclinical	Qu et al. (2023) *A. muciniphila*; Amuc_1100	HFD-induced NAFLD mice	Reduced aminotransferases and hepatic inflammatory signaling and improved steatosis-related measures.	—	Preventive mouse model; direct hepatic target engagement not shown.
Liver preclinical	Cheng et al. (2024) Pasteurized cells; Amuc_1100	Alcohol-associated liver injury mice	Pasteurized cells improved liver injury and lipid-metabolism readouts; Amuc_1100 partly recapitulated protection.	—	Single model; no exposure matching or loss-of-function test.
Liver preclinical	Zhuge et al. (2025) *A. muciniphila*-associated acetate	HFHFD mice; hepatocyte systems	Acetate activated hepatic AMPK/SIRT1/PGC-1α signaling and reduced ferroptosis and lipid peroxidation.	—	Supports a metabolite mechanism beyond the protein/EV framework.
Liver preclinical	Kwak et al. (2026) *A. muciniphila* EVs	Comparative microbial EV study; *in vitro* MASH model	Reduced lipid accumulation and expression of lipogenesis-related genes.	—	PEG precipitation may co-isolate soluble or non-vesicular material.
Liver preclinical	Li J. et al. (2026) L-norleucine	Molecular and metabolic models	Linked an *A. muciniphila*-associated metabolite to FABP1-dependent fatty-acid transport.	—	New effector; contribution to pasteurized whole-cell efficacy is unknown.
Human completed	Depommier et al. (2019) Live or pasteurized MucT (10^10^ cells/day, oral)	Exploratory double-blind placebo-controlled pilot study; n = 40 enrolled (32 completed); 3 months (12 weeks)	Primary: safety/tolerability and metabolic parameters — pasteurized improved insulin sensitivity (+28.6%, P = 0.002) and reduced insulinemia and total cholesterol vs placebo (prespecified); live MucT did not significantly improve insulin sensitivity. Liver: serum enzymes only (safety outcome), no histology or MASLD endpoint.	Academic (UCLouvain; Baillet Latour/Walloon Region/ERC/FNRS); P.D. Cani and W.M. de Vos are co-founders of A-Mansia Biotech SA; five authors are patent inventors.	Small study; liver histology and effector exposure were not assessed.
Human completed	Mount et al. (2026) Pasteurized MucT (3 × 10^10^ heat-treated cells/day, oral capsule)	Randomized placebo-controlled trial; n = 90; weight-maintenance phase (∼6 months)	Primary: body-weight regain during weight maintenance — reduced vs placebo (prespecified). Liver: serum enzymes only (secondary), no histology or MASLD endpoint.	Academic-led (Maastricht UMC); industry-funded by The Akkermansia Company SA; authors incl. co-founder/CTO, CMO, employee, scientific advisor, and patent inventors.	Metabolic rather than liver-directed endpoint; one active dose.
Human completed	Suenaert et al. (2026) Pasteurized MucT (3 × 10^10^ cells/day, oral capsule)	Multicenter double-blind RCT; n = 143 randomized (142 analyzed); 16 weeks (4 months)	Primary: whole-body insulin sensitivity (Matsuda index) at 16 weeks — not met in ITT (prespecified); benefit in the median-split low-baseline-*Akkermansia* subgroup (∼35 per arm) was *post hoc*/exploratory. Liver: serum enzymes only (secondary), no histology or MASLD endpoint.	Industry (The Akkermansia Company/A-Mansia Biotech S.A.); multiple authors are employees/consultants/co-founders/patent inventors.	Subgroup findings require prospective validation; no effector assay.
Registered ongoing	NCT06873165 Pasteurized NTUH_Amuc03 (LWHK0003); fixed-dose oral capsule (dose not specified in registration)	Triple-masked, randomized, fixed-dose vs placebo; estimated n = 40; 3-month intervention (primary readouts at 12–16 weeks); phase not specified; estimated study completion December 2026	Primary outcomes: gut microbiota (16S rRNA) and changes in liver stiffness (FibroScan) and non-invasive fibrosis markers (FIB-4)	Sponsor: National Taiwan University Hospital	No prespecified Amuc_1100, P9, or AmEV endpoint.
Registered ongoing	NCT05821010 Augustijn et al. (2025) Pasteurized *A. muciniphila* within synbiotic/FMT regimen; oral	Phase 2, quadruple-masked; estimated n = 48; 24 weeks; estimated study completion August 2026	Primary: liver histology (prespecified); broad secondary metabolic, immune, imaging, and microbiome outcomes	Sponsor: Amsterdam UMC	Active arm combines pasteurized *A. muciniphila* with lyophilized FMT, other probiotics (*A. soehngenii*, *B. animalis* subsp. *lactis*), and fructo-oligosaccharides, so the independent contribution of *A. muciniphila* cannot be determined. Estimated completion date may lag actual progress.
Registered ongoing	NCT07488975 Pasteurized LWHK0003 (NTUH_Amuc03); oral capsule	Phase 1, triple-masked, dose-ranging (10^9^/10^10^/10^11^ CFU); estimated n = 40; primary readouts at 12 and 16 weeks (treatment up to 52 weeks); estimated study completion December 2026	Outcomes: hepatic steatosis, liver stiffness (FibroScan/MRE), and non-invasive fibrosis markers (FIB-4) across three dose levels	Sponsor: Leeuwenhoek Laboratories Co. Ltd. (industry)	No prespecified defined-effector exposure endpoint.

## 2 Evidence landscape: pasteurized *Akkermansia muciniphila* as a multi-effector system

Three development streams coexist: live biotherapeutics, whose activity depends on viable colonization; pasteurized whole-cell postbiotics with improved stability and a reproducible metabolic record; and isolated effectors with conventional target-based pharmacology. The field has advanced furthest clinically with pasteurized cells, mechanistically with Amuc_1100 and P9, and in systemic delivery with AmEVs, yet none has established a validated bridge from molecular exposure to human liver response.

Across this evidence, effectors fall into three evidentiary tiers. (1) Amuc_1100 is demonstrably retained and active after pasteurization (heat stability; recapitulation by pasteurized cells). (2) P9 and AmEVs are produced by live *A. muciniphila* and characterized in isolated or enriched form, but their retention in pasteurized products is unestablished. (3) Membrane lipids and diffusible metabolites (acetate, propionate, L-norleucine) are *A. muciniphila*-associated cell-free products whose contribution to pasteurized efficacy is undemonstrated. We therefore treat the multi-effector system as a testable development model, not an established property of every product.

### 2.1 Context dependence of whole-cell efficacy

The foundational mouse study showed that *A. muciniphila* reversed high-fat-diet-induced metabolic dysfunction and restored gut-barrier features, consistent with direct adhesion and epithelial-integrity experiments (Everard et al., 2013; Reunanen et al., 2015). Plovier et al. then reported that pasteurization at 70 °C for 30 min enhanced selected metabolic effects in obese mice and identified the outer-membrane protein Amuc_1100 as a heat-stable contributor; subsequent receptor assays supported TLR2-dependent barrier and immune activity (Plovier et al., 2017; Ottman et al., 2017). Pasteurized MucT also increased energy expenditure and fecal energy loss in diet-induced obese mice, illustrating that whole-body outcomes can arise through mechanisms beyond a single hepatic target (Depommier et al., 2020). Pasteurized Akk11 has outperformed its live counterpart for selected obesity and intestinal outcomes in mice, but this should not be generalized across strains, doses, models, or endpoints (Feng et al., 2025). Pasteurized *A. muciniphila* is therefore a product class rather than a uniform entity.

Human evidence is encouraging but not uniform. Observational intervention data first linked higher baseline *A. muciniphila* abundance to more favorable metabolic responses during calorie restriction (Dao et al., 2016). In 40 overweight or obese insulin-resistant adults, daily pasteurized *A. muciniphila* for 3 months improved insulin sensitivity and several metabolic measures, whereas the live preparation did not significantly improve insulin sensitivity (Depommier et al., 2019). A randomized study of 90 adults reported less weight regain after a low-energy diet with pasteurized MucT than with placebo (Mount et al., 2026). A separate trial in overweight or obese adults with type 2 diabetes found no overall between-group efficacy but again suggested greater benefit among participants with low baseline abundance (Zhang et al., 2025). Similarly, a 143-participant multicenter trial did not meet its primary whole-body insulin-sensitivity endpoint (Matsuda index at 16 weeks) in the intention-to-treat population; exploratory *post hoc* subgroup analyses of a median-split subgroup (∼35 participants per arm), without a prespecified multiplicity procedure, suggested benefits in participants with low baseline intestinal *Akkermansia* abundance (Suenaert et al., 2026). These studies support biological activity and short-term tolerability, but they do not establish a general liver indication, a universal superiority of pasteurized over live bacteria, or a molecular exposure-response relationship. The completed trials measured metabolic and weight-related outcomes rather than liver histology or validated MASLD efficacy endpoints.

### 2.2 Direct and indirect liver-directed evidence

Earlier interpretations of liver benefit relied heavily on indirect, whole-body readouts—body weight, insulin sensitivity, and intestinal barrier or permeability function. Direct liver-directed experiments (hepatic steatosis, inflammation, and fibrosis markers) now provide a stronger foundation. Live *A. muciniphila* reduced hepatic triglyceride synthesis and inflammation in diet-induced fatty liver, while another study linked its benefit to reshaped gut microbiota and bile-acid metabolism (Kim et al., 2020; Wu et al., 2023). In high-fat-diet-fed mice, both *A. muciniphila* and purified Amuc_1100 reduced serum aminotransferases, hepatic inflammatory signaling, and intestinal permeability while improving steatosis-related measures (Qu et al., 2023). In alcohol-associated liver disease, restoration of *A. muciniphila* improved barrier integrity and liver injury, and later work showed that pasteurized bacteria improved hepatic histology and lipid-metabolism gene expression while heterologously expressed Amuc_1100 partly recapitulated these effects (Grander et al., 2018; Cheng et al., 2024). A separate cirrhosis model linked small-intestinal *A. muciniphila* administration to improved fibrosis and intestinal barrier features (Oguri et al., 2024). These studies strengthen biological plausibility, although they remain preventive or early-treatment animal experiments rather than clinical pharmacology.

AmEVs provide a second liver-relevant route. Chelakkot et al. showed that orally administered AmEVs enhance intestinal tight-junction integrity through AMPK-associated regulation of occludin, and an independent obesity study found that AmEV administration improved barrier, inflammatory, and metabolic readouts in mice (Chelakkot et al., 2018; Ashrafian et al., 2019). In an HFD/CCl_4_ model, live bacteria, pasteurized bacteria, and AmEV-containing preparations improved permeability, inflammation, histopathology, and fibrosis-related markers; AmEVs also suppressed activation-related responses in LX-2 hepatic stellate cells (Keshavarz Azizi Raftar et al., 2021). More recently, a comparative gut-microbial-EV study found that *A. muciniphila* EVs reduced lipid accumulation and lipogenic gene expression in an *in vitro* MASH system, although PEG precipitation may have co-isolated non-vesicular material (Kwak et al., 2026). Together, these data support liver relevance but do not yet demonstrate which AmEV cargo reaches hepatocytes, Kupffer cells, or stellate cells at an active concentration.

The expanding metabolite literature further changes the pharmacological picture. These small molecules are not unique to *A. muciniphila*—acetate and propionate are common gut-microbial fermentation products—so attribution to this bacterium rests on specific experimental evidence rather than on the metabolite itself. Acetate was linked to *A. muciniphila* by germ-free mono-association and treatment-related enrichment, and was traced to hepatic AMP generation and AMPK/SIRT1/PGC-1α activation that reduced lipid peroxidation and ferroptosis in a diet-induced fatty-liver model (Zhuge et al., 2025). Propionate was elevated after *A. muciniphila* administration mainly by enriching propionate-producing taxa and intestinal transport rather than by direct secretion (Zhang et al., 2026). Microbiota-derived α-ketoisovaleric acid has also been connected to hepatic PI3K/Akt signaling, while *A. muciniphila*-associated L-norleucine, identified by metabolite enrichment coupled with untargeted metabolomics, modulated FABP1-dependent fatty-acid transport (Liu et al., 2025; Li J. et al., 2026). As with the protein effectors, these hepatic signaling changes are pathway-associated readouts rather than proof of direct hepatic target engagement at a defined exposure. These studies do not displace Amuc_1100, P9, or AmEVs; instead, they show that pasteurized or cell-free activity may arise from a broader and preparation-dependent effector repertoire.

### 2.3 Complementary effector classes and mechanistic reach

Amuc_1100 is the most mature protein effector. It activates TLR2-associated host responses, improves epithelial-barrier function, and partly reproduces whole-cell metabolic activity (Plovier et al., 2017). Structural analysis shows a stable oligomeric architecture compatible with persistence after pasteurization (Wang et al., 2020). Its liver-directed activity is supported by the NAFLD and alcohol-associated liver disease studies above, but oral bioavailability, tissue exposure, target occupancy, and pharmacokinetics remain undefined. Apparent downstream changes in hepatic TLR4/NF-κB/NLRP3 signaling should therefore be interpreted as pathway-associated outcomes rather than proof that orally administered Amuc_1100 directly engages a hepatic receptor.

P9 is mechanistically distinct. This secreted 84-kDa protein binds ICAM-2 on enteroendocrine L cells, activates PLC-Ca^2+^-CREB signaling, increases GLP-1 secretion, and improves metabolic phenotypes in high-fat-diet-fed mice (Yoon et al., 2021). Engineered *Lactococcus lactis* can deliver biologically active P9, confirming that heterologous expression is feasible (Di et al., 2025). Its relevance to MASLD is indirect: endogenous GLP-1 could influence weight, insulin sensitivity, and hepatic substrate flux, but purified P9 has not been tested against histological MASH or fibrosis endpoints, and whether P9 remains quantitatively active in a pasteurized whole-cell preparation is unestablished.

AmEVs are intrinsically multi-component effectors rather than single molecules. They can carry proteins, lipids, nucleic acids, and metabolites, and their activity depends on culture conditions, isolation method, normalization strategy, and storage. Their intestinal barrier effects are established more directly than their systemic liver actions (Chelakkot et al., 2018; Wang et al., 2025). A chemically defined *A. muciniphila* membrane phospholipid, a15:0-i15:0 phosphatidylethanolamine, signals through a TLR2-TLR1 heterodimer (Bae et al., 2022), while a distinct lipooligosaccharide engages TLR2 and TLR4 (Garcia-Vello et al., 2024). Neither finding by itself proves that the lipid is an active AmEV cargo in the liver. The appropriate conclusion is that AmEVs are promising delivery structures whose active cargo and hepatic cellular targets remain incompletely resolved.

Figure 1 summarizes how Amuc_1100, P9, and AmEVs may connect intestinal events to liver responses. The receptor-level intestinal mechanisms are supported more directly than several portal and hepatic steps, whose evidentiary limits are specified in the legend (Figure 1).

**FIGURE 1:**
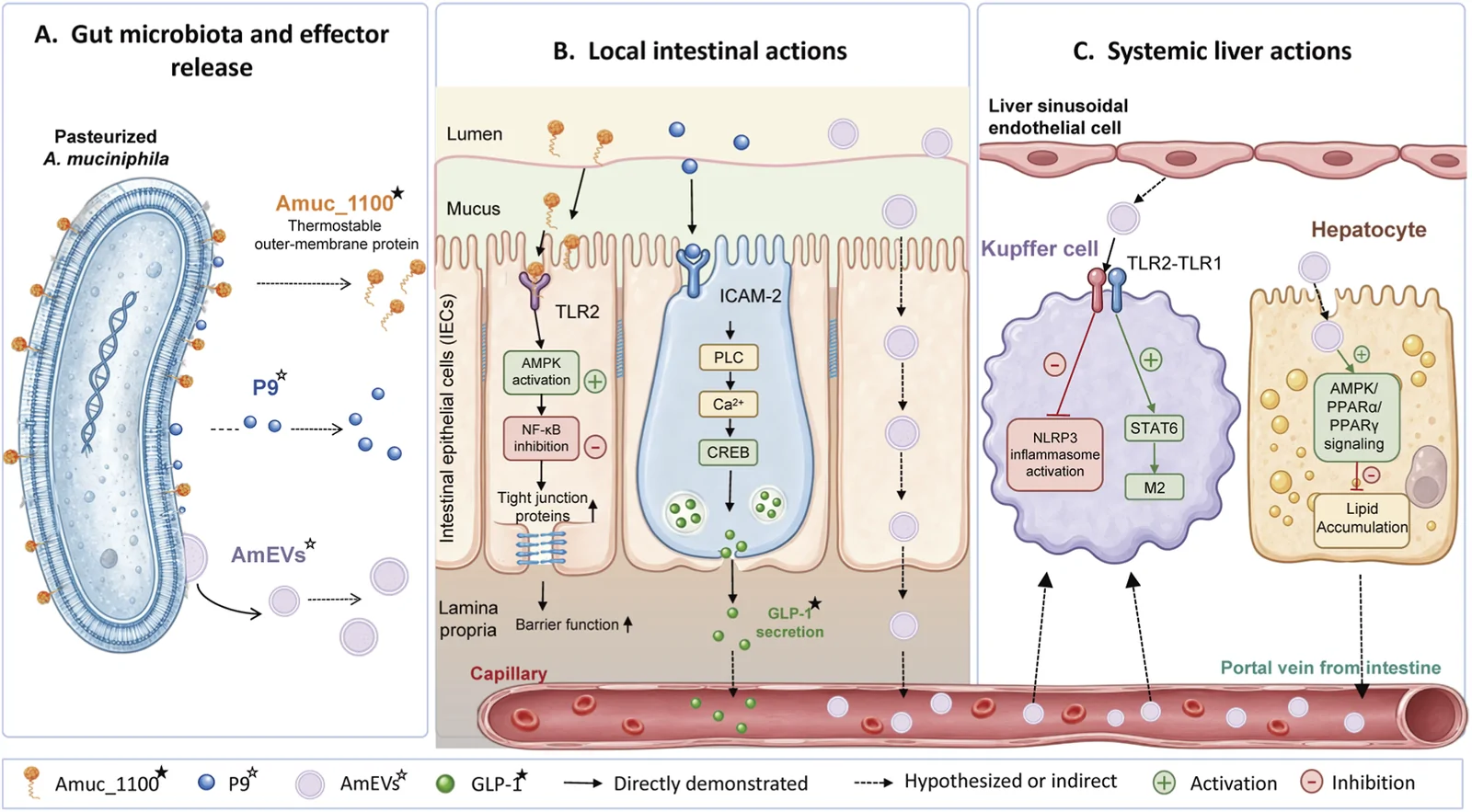
Integrative model of selected *Akkermansia muciniphila*-associated effectors across the gut-liver axis. **(A)** Effector molecules associated with *A. muciniphila*. **(B)** Local intestinal actions: Amuc_1100 strengthens intestinal epithelial barrier function via TLR2-dependent signalling (AMPK activation; NF-κB inhibition) in intestinal epithelial cells (IECs). P9 binds ICAM-2 on enteroendocrine L cells and triggers GLP-1 secretion through the PLC-Ca^2+^-CREB cascade. AmEVs are proposed to traverse the intestinal epithelium and enter portal circulation. **(C)** Systemic liver actions: AmEVs may reach the liver via the portal vein and act on Kupffer cells and hepatocytes. Panel C represents a hypothesis-generating synthesis instead of a fully validated linear pathway; direct delivery of AmEVs to distinct hepatic cell types, TLR2-TLR1-activating cargo within AmEVs, and the sequential signalling events illustrated in Kupffer cells and hepatocytes have not been jointly experimentally verified. Solid arrows indicate regulatory steps supported by direct experimental evidence; dashed arrows represent hypothesized, indirect, or long-distance transport processes. Symbols: ★, effectors or downstream outputs validated in pasteurized whole-cell *A. muciniphila* preparations (Amuc_1100, GLP-1); ☆, *A. muciniphila*-derived effectors characterized in isolated systems, whose functional persistence in pasteurized whole-cell preparations remains unconfirmed (P9, AmEVs). Green plus symbols denote activation; red minus symbols denote inhibition. Abbreviations: AmEVs, *A. muciniphila*-derived extracellular vesicles; AMPK, AMP-activated protein kinase; CREB, cAMP response element-binding protein; GLP-1, glucagon-like peptide-1; ICAM-2, intercellular adhesion molecule 2; IECs, intestinal epithelial cells; M2, alternatively activated macrophage phenotype; NF-κB, nuclear factor kappa B; NLRP3, NOD-like receptor family pyrin domain-containing protein 3; PLC, phospholipase C; PPARα, peroxisome proliferator-activated receptor alpha; PPARγ, peroxisome proliferator-activated receptor gamma; STAT6, signal transducer and activator of transcription 6; TLR2/TLR1, Toll-like receptor 2/Toll-like receptor 1 heterodimer.

### 2.4 Clinical translation of whole-cell preparations

No published human study administered purified Amuc_1100, P9, or AmEVs, and registered liver-directed programs remain whole-cell based. NCT06873165 and NCT07488975 are pasteurized whole-cell trials of the same strain—NTUH_Amuc03, also designated LWHK0003 — conducted at the same center (National Taiwan University Hospital, Taipei) with the same target enrollment (n = 40) and overlapping dates; they are a fixed-dose two-arm trial and a phase 1 three-dose trial, respectively, and may represent closely related or overlapping registrations of a single development program rather than fully independent studies. NCT05821010 combines pasteurized *A. muciniphila* with synbiotics and fecal microbiota transplantation in MASH (Augustijn et al., 2025). None prespecifies a validated Amuc_1100, P9, or AmEV exposure assay; the landscape is summarized in Table 1.

## 3 Unresolved causality, exposure, and product definition

The central gap is no longer whether pasteurized *A. muciniphila* can be biologically active. It is whether its activity can be converted into a causal, measurable, and transferable pharmacological model.

### 3.1 Lack of quantitative effector deconvolution

Most studies establish effector sufficiency, whereas few have tested necessity. Purified Amuc_1100, recombinant P9, isolated AmEVs, and selected metabolites each produce phenotypes, but in different experimental systems. No experiment has quantified their contributions within a single pasteurized preparation. Their interactions may be additive, redundant, antagonistic, or sequential, and pasteurization may change molecular accessibility. An effector can be sufficient at an experimental dose yet contribute little at its endogenous abundance. Conversely, a modest component may become essential through synergy or spatial presentation on the bacterial surface.

This asymmetry also constrains the gut-liver sequence: Amuc_1100 and P9 have direct intestinal receptor-level support, whereas the systemic AmEV route combines biodistribution, hepatocyte, stellate-cell, and immune observations from different systems and remains a testable model rather than one continuous causal chain.

### 3.2 Absence of human effector pharmacokinetics

Whole-cell dose is usually expressed as CFU equivalents or total cells before inactivation. That unit does not report the exposure-relevant attributes of the key effector classes—Amuc_1100, P9, AmEVs, and small-molecule effectors. Two lots with the same nominal cell dose may therefore deliver different pharmacological exposures. This problem is analogous to other complex biological products in which identity, content, potency, and stability require orthogonal assays rather than one mass or count measurement (Wu C. et al., 2020).

The available human trials also prioritize metabolic or histological outcomes over mechanism. This is appropriate for early efficacy evaluation, but it prevents learning why participants respond differently. Retrospective analyses are possible only if serum, stool, and product lots have been stored under conditions compatible with protein, vesicle, and metabolite assays.

### 3.3 Strain identity and processing as pharmacological variables

Marked strain-level efficacy differences have been observed in cell and animal screens (Li H. et al., 2026; Yang et al., 2020). Genomic diversity may alter secreted proteins, surface architecture, vesicle production, mucin utilization, and metabolite output (Molteni et al., 2025; Mueller et al., 2024). Culture medium, growth phase, oxygen exposure, heat profile, washing, formulation, and storage can further change the final product. Sequence identity of Amuc_1100 alone cannot establish equivalence when expression abundance, oligomerization, membrane presentation, and co-effectors differ. Product development therefore needs a strain-process-effector chain of comparability, not a species label.

Regulatory precedent supports this direction without equating a postbiotic with a botanical drug. FDA-authored analysis of complex botanical development emphasizes chemical characterization, manufacturing control, biological assays, and consistency when no single active constituent explains the product (Wu C. et al., 2020). EFSA safety evaluation and postbiotic guidance likewise link safety, identity, and intended use to a defined preparation rather than to a species name (EFSA NDA Panel (EFSA Panel on Nutrition, Novel Foods and Food Allergens) et al., 2025; Vinderola et al., 2024).

### 3.4 Boundary conditions and alternative interpretations

A reductionist program should not assume that isolating an effector will automatically produce a better therapy. Pasteurized cells preserve spatial organization, repeated surface epitopes, membrane lipids, vesicles, enzymes, and small molecules in a format that may generate coordinated barrier, endocrine, and immune effects. This complexity may be an advantage for multifactorial metabolic disease, particularly when modest actions across several pathways are safer than maximal activation of one target. The broader probiotic literature also warns that efficacy is context dependent and that a mechanistically attractive organism may behave differently across hosts, diets, background microbiota, and disease stages (Suez et al., 2019). Deconvolution should therefore identify the sources of efficacy and variability, not predetermine that a purified component must replace the parent preparation.

Conversely, a whole-cell product is not adequately characterized simply because it is non-viable. Heat inactivation prevents replication but not lot-to-lot variation, innate immune activity, or changes in molecular accessibility. Product complexity complicates dose selection, pharmacokinetic interpretation, and comparison across trials. A food or broad metabolic-health product may rely on compositional and functional specifications, whereas a prescription claim for MASH resolution or fibrosis improvement demands a stronger link among dose, potency, mechanism, and clinical response. The appropriate evidentiary standard should therefore reflect both product format and intended claim.

Disease-stage selection is another boundary condition. Improvements in permeability, insulin sensitivity, or hepatic lipid accumulation may matter most in early MASLD, while advanced MASH and fibrosis involve established immune and stromal programs that may not respond to the same intervention. The Raftar study provides proof of concept in a combined HFD/CCl_4_ injury model, but preventive co-treatment is not equivalent to reversing established fibrosis (Keshavarz Azizi Raftar et al., 2021). Future studies should distinguish prevention from treatment, simple steatosis from inflammatory MASH, and fibrosis regression from reduced injury. Sex, diet, baseline *Akkermansia* abundance, and weight loss are biological modifiers, not experimental noise.

## 4 A testable pharmacological roadmap

### 4.1 Orthogonal effector deconvolution

The highest-value experiment is a matched deconvolution study using one strain, one manufacturing process, and one disease model. A pasteurized reference lot should be compared with preparations selectively depleted of surface Amuc_1100, soluble P9, or vesicles; purified add-back arms; a reconstituted effector combination; and vehicle controls. Genetic loss-of-function and complementation would offer strong causal inference if a robust anaerobic engineering system is available. Where it is not, immunodepletion, affinity capture, proteolytic accessibility mapping, vesicle-depletion/reconstitution, and size- or polarity-based fractionation provide immediately feasible orthogonal perturbations. Agreement across two independent perturbation methods would be more persuasive than reliance on a single engineered strain.

The study should quantify the actual effector dose in every arm and include both intestinal and hepatic endpoints. Intestinal readouts should include permeability, tight-junction proteins, inflammatory signaling, and GLP-1. Liver readouts should include triglyceride content, histological steatosis and inflammation, fibrosis markers when appropriate, and pathway-specific measurements linked to the proposed effector. A factorial design can test interactions among Amuc_1100, P9, and AmEVs; adding a metabolite-rich fraction would test whether the three leading classes leave substantial residual activity. Predefined equivalence or non-inferiority margins are preferable to interpreting a series of pairwise P values.

One discovery model is insufficient for a development decision. The most informative sequence would use an obesity-associated steatosis model for metabolic efficacy, followed by an established MASH or fibrosis model for hepatic validation. Both sexes should be included unless a justified sex-specific mechanism is being tested. Pair-feeding or weight-matched controls are essential when a treatment changes food intake or body mass, because reduced steatosis may otherwise be misassigned to a direct hepatic mechanism. Tissue-distribution studies with labeled or molecularly traceable effectors should determine whether the relevant action is confined to the gut, mediated by endocrine or metabolite signals, or requires portal and hepatic exposure.

### 4.2 Effector-aware clinical pharmacology

Ongoing and future whole-cell trials should retain product lots and longitudinal biospecimens and prespecify exploratory exposure-response analyses. Candidate assays include targeted proteomics or immunoassays for Amuc_1100 and P9, nanoparticle and cargo profiling for AmEVs, and targeted metabolomics for acetate, propionate, L-norleucine, and other strain-linked molecules. Assays must distinguish administered product from endogenous *Akkermansia* and should report lower limits of quantification, recovery, stability, and inter-lot variability. A composite effector fingerprint may prove more informative than any single analyte.

These measurements could determine whether nominal dose predicts molecular exposure, whether exposure varies with baseline microbiota, whether liver response is mediated by barrier restoration or systemic effector delivery, and whether non-response reflects inadequate exposure or biological resistance—without requiring an isolated effector trial at the outset. The registered dose-ranging studies are especially suitable for this logic, but retrospective work should be described as exploratory unless sampling and analysis were prespecified.

Clinical endpoints should follow an explicit hierarchy. Early trials can use safety, tolerability, product exposure, MRI-based liver fat, aminotransferases, and validated non-invasive fibrosis measures to establish pharmacological activity. Histological MASH resolution or fibrosis improvement should be reserved for adequately powered studies in appropriately staged disease. Mechanistic biomarkers should be selected before unblinding and analyzed alongside changes in body weight and glycemic control so that liver improvement is not automatically labeled a direct hepatic effect. A minimal translational dataset would connect product lot, administered dose, effector fingerprint, host exposure, intermediate mechanism, and liver outcome for each participant.

### 4.3 Evidence-based product selection

Three development formats are plausible. A standardized pasteurized whole-cell product preserves multi-effector activity and may be optimal when synergy, manufacturing simplicity, and nutritional or broad metabolic use are priorities. An effector-standardized whole-cell product would retain that complexity while adding release specifications for key proteins, vesicles, and potency. A purified or recombinant effector offers clearer pharmacokinetics and target attribution but may lose cooperative activity and face oral-delivery challenges, particularly for proteins (Brown et al., 2019). The deconvolution and exposure data should determine which format is justified for each indication.

For the intermediate effector-standardized format, a practical release panel could combine strain identity by whole-genome sequencing; quantitative Amuc_1100 and P9 assays; vesicle abundance, size distribution, and selected cargo markers; targeted metabolites; residual viability; and one or more cell-based potency assays. Stability studies should test whether these attributes remain within predefined ranges throughout storage. Correlation of release attributes with *in vivo* or clinical response would then allow a smaller set of critical quality attributes to replace an indefinitely expanding analytical panel. This approach is consistent with integrated postbiotic development, in which manufacturing control and biological function are developed together rather than sequentially (Chikindas et al., 2026).

Combination therapy is a specific test case. P9 stimulates endogenous GLP-1, while GLP-1 receptor agonists improve weight and liver-related outcomes through systemic pharmacology. Preclinical combination of a GLP-1 receptor agonist with *A. muciniphila* Akk11 improved adiposity and MASLD-related measures in diabetic mice (Gao et al., 2026). This supports factorial testing of whole cells, P9 or P9-enriched preparations, and a GLP-1 receptor agonist, with food intake and weight-matched controls. The aim should be to identify additivity, redundancy, or a dose-sparing effect, not to presume a positive-feedback loop.

Safety and manufacturability must be integrated from the beginning. Available toxicology studies support the safety of tested live and pasteurized preparations at high doses, including a separate multidomain assessment of pasteurized YGMCC2645 (Yu et al., 2024; Druart et al., 2021; Tian et al., 2026), but purified proteins and vesicles require their own impurity, immunogenicity, biodistribution, and stability programs. For AmEVs, isolation method and residual free protein or endotoxin-like activity are part of product identity. For recombinant effectors, proteolysis and low intestinal permeability make delivery strategy a core pharmacological variable rather than a downstream formulation detail.

## 5 Conclusion

Pasteurized *A. muciniphila* is neither a conventional single-molecule drug nor an indivisible biomass, but a measurable multi-effector pharmacological system. Liver studies support relevance to steatosis, inflammation, ferroptosis, and fibrosis, but the evidence remains predominantly preclinical. Completed human trials assessed metabolic and weight-related outcomes rather than liver histology or validated MASLD efficacy endpoints. Accordingly, the current human evidence does not establish clinical efficacy for MASLD, MASH, or liver fibrosis. Amuc_1100, P9, and AmEVs provide useful mechanistic anchors, although recent metabolite studies show that they cannot explain the full activity of every preparation.

Progress now depends on linking product composition with exposure, mechanism, and liver outcome. Orthogonal deconvolution, effector-aware clinical sampling, and strain-process-potency specifications would make those links testable. The evidence may favor a pasteurized whole-cell product for one indication and an effector-standardized preparation or purified molecule for another. Product format should follow those results rather than precede them.
